# Breakers Ahead

**Published:** 1908-01

**Authors:** 


### EDITORIAL DEPARTMENT.

### BREAKERS AHEAD.

THE "RED BACK" FLASHES THE DANGER SIGNAL.

Our State Medical Association is a grand body [illegible] workers in the cause of humanity. It is an honor to the profession and a credit to Texas. The physicians of the State accord it a loyal support, and are proud of its *cohesion* and virility. The more so because, under the old organization, it lacked both. It had a precarious existence for many years, and crumbling and disintegration characterized it. It exemplified constructive and destructive metabolism. As fast as it would build up it would crumble; so that in 1884 there were 280 members and in 1903 there were 380, or a net gain of only 100 in nineteen years. Meantime the record shows that some 800 or more new members were admitted in that time, and nearly as many dropped out; some from indifference, and failure to pay dues; some because of dissensions, and a downward tendency in the matter of ethics as regards irregulars, and some because of a pronounced tendency to drift into politics.

The membership now numbers 3000, in round numbers, and it is constantly increasing. I am not aware that it has lost a member (except by death) in the four years since reorganization. In that time it has done much excellent work, too recent and too well known to here recount. The success of its official organ, too, has been most gratifying, and, unless we drift onto the shoals and rocks which wrecked the old boat, the prospect is far fair sailing and continued progress and usefulness.

*There is the danger.* There is loud and outspoken objection to the policy of the House of Delegates in the matter of affiliation with the quacks; but, so far as I have been able to learn, there is a disposition to accept the One Board Bill because it is *an Association measure,* and to sink personal objections in deference to the will of the majority, and for the sake of peace and harmony. The writer vigorously opposed it, believing—as he still believes—that it was an error of judgment; that zeal got the better of wisdom, and led the House to extremes in the matter of reform,—in order to *stand in with* the great medico-political machine in Chicago.

The general sentiment amongst the older members (and I have many and emphatic letters from some of the "fathers") is that it is a *step backwards,* a retrocession, much to be deplored; and all deprecate and protest against the tendency recently shown, to *drift into politics.* This is the rock upon which we will split, unless wise counsels prevail. The Association must be kept out of politics. It will drive off men now staunch and loyal, and ruin our splendid organization. I have heard expressions (and others have heard them) about like this: "If the Association is to become a political machine, I have attended my last meeting."

In last issue I called attention to this matter and cited an Associated Press dispatch from San Antonio to the effect that the press correspondent had the authority of "one high in medical councils" for saying that the State Medical Association would boycott Davidson and support Looney for Attorney General. No name was called, but the Councilor of the Fifth (San Antonio) District took it to himself, and published a general denial. If he "didn't say it," how did he know that he was meant? It becomes now a question of veracity with him and the correspondent, and we will let it go at that. And we here express the hope that the House of Delegates will promptly repudiate any tendency to commit it as a body to the candidacy of anyone; and demand an avoidance of the subject as foreign to the objects and purposes of the Association and dangerous to its peace, and to its very existence.

In the meantime I had received strong letters from leading men, all over the State, with permission to publish them, protesting against the tendency to politics. But I will suppress them, in the hope that we will hear no more of boycotting one candidate and "whooping 'em up" for another.

I had written the above when I was surprised and chagrined to see that the December number of the Association's official organ—*The Texas State Journal of Medicine*—takes up the subject and openly advocates Mr. Looney's election, enumerating, as he would do from the stump, his actual or supposed claims to the support of the doctors. The editorial closes with: "Hats Off For Looney."

The founders of the *Journal,* its trustees and readers, must have read this, as we did, with surprise and regret. It is not only committing the Association to politics,—it is tantamount to a declaration that it will *take a hand* in the next election, but it is partizan politics, and will be resented. It is calculated to create a split, inasmuch as Senator Bailey and Mr. Davidson have, each, warm partizans and supporters amongst our membership, as well as strong opponents; and it is generally understood that these gentlemen represent the opposing factions in the issue that will overshadow all others in the coming election, and, that Senator Looney is enlisted under the Bailey banner. The members of the Association will not submit to dictation by the editor, nor approve of the policy of the Association organ in such a radical departure from the purposes for which it was founded. They will exercise the right to vote as they please.

The Association must be steered clear of Scylla on the one side and Charybdis on the other, or it will surely founder. Selah!

